# The Safety, Tolerability, Pharmacokinetics, and Clinical Efficacy of the NLRX1 agonist NX-13 in Active Ulcerative Colitis: Results of a Phase 1b Study

**DOI:** 10.1093/ecco-jcc/jjad192

**Published:** 2023-11-11

**Authors:** Bram Verstockt, Severine Vermeire, Laurent Peyrin-Biroulet, Rebecca Mosig, Brian G Feagan, Jean-Frederic Colombel, Britta Siegmund, Florian Rieder, Stefan Schreiber, Andres Yarur, Remo Panaccione, Marla Dubinsky, Simon Lichtiger, Fabio Cataldi, Silvio Danese

**Affiliations:** Department of Gastroenterology and Hepatology, University Hospitals Leuven, KU Leuven, Leuven, Belgium; Department of Chronic Diseases and Metabolism, KU Leuven, Leuven, Belgium; Department of Gastroenterology and Hepatology, University Hospitals Leuven, KU Leuven, Leuven, Belgium; Department of Chronic Diseases and Metabolism, KU Leuven, Leuven, Belgium; Department of Gastroenterology, Nancy University Hospital, F-54500 Vandœuvre-lès-Nancy, France; INSERM, NGERE, University of Lorraine, F-54000 Nancy, France; INFINY Institute, Nancy University Hospital, F-54500 Vandœuvre-lès-Nancy, France; FHU-CURE, Nancy University Hospital, F-54500 Vandœuvre-lès-Nancy, France; Groupe Hospitalier privé Ambroise Paré – Hartmann, Paris IBD Center, 92200 Neuilly sur Seine, France; Division of Gastroenterology and Hepatology, McGill University Health Centre, Montreal, Quebec, Canada; Landos Biopharma, Blacksburg, VA, USA; Department of Medicine, Division of Gastroenterology, University of Western Ontario, London, Ontario, Canada; Alimentiv Inc, London, Ontario, Canada; Department of Epidemiology and Biostatistics, Western University, London, Ontario, Canada; The Dr. Henry D. Janowitz Division of Gastroenterology, Icahn School of Medicine at Mount Sinai, New York, NY, USA; Division of Gastroenterology, Infectiology and Rheumatology, Charité – Universitätsmedizin Berlin, corporate member of Freie Universität Berlin and Humboldt-Universität zu Berlin, Berlin, Germany; Department of Gastroenterology, Hepatology, and Nutrition, Digestive Disease and Surgery Institute, Cleveland Clinic, Cleveland, OH, USA; Institute of Clinical Molecular Biology, Christian-Albrechts-University and University Hospital Schleswig-Holstein, Campus Kiel, Kiel, Germany; Department of Internal Medicine, Christian-Albrechts-University and University Hospital Schleswig-Holstein, Campus Kiel, Kiel, Germany; Division of Gastroenterology and Hepatology, Center for Inflammatory Bowel Diseases. Cedars Sinai Medical Center, Los Angeles, CA, USA; Division of Gastroenterology & Hepatology, Department of Medicine, Cumming School of Medicine, University of Calgary, Calgary, Alberta, Canada; Division of Pediatric Gastroenterology and Nutrition, Mount Sinai Kravis Children’s Hospital, Icahn School of Medicine Mount Sinai, New York, NY, USA; Landos Biopharma, Blacksburg, VA, USA; Landos Biopharma, Blacksburg, VA, USA; Gastroenterology and Gastrointestinal Endoscopy Unit, IRCCS San Raffaele Scientific Institute, Vita-Salute San Raffaele University, Milan, Italy

**Keywords:** Randomized controlled clinical trial, NLRX1, NX-13, immunometabolism, novel mechanism of action, NEXUS, phase 1b

## Abstract

**Background and Aims:**

NX-13 activation of NLRX1 reduces intracellular reactive oxygen species and decreases inflammation in animal models of colitis. A phase 1a trial demonstrated a gut-selective pharmacokinetic profile with good tolerability. This phase Ib study aimed to evaluate the safety, tolerability, and pharmacokinetics of NX-13 in patients with active ulcerative colitis [UC].

**Methods:**

We conducted a multicentre, randomized, double-blind, placebo-controlled trial of NX-13 in patients with active UC. Patients with a Mayo Clinic Score of 4–10 were randomly assigned [3:3:3:1 ratio] to three NX-13 oral dose groups (250 mg immediate release [IR], 500 mg IR, or 500 mg delayed release [DR], or placebo) once daily for 4 weeks. Safety and pharmacokinetics were the primary and secondary objectives, respectively.

**Results:**

Thirty-eight patients [11 females] were recruited and randomized to placebo [five], NX-13 250 mg IR [11], NX-13 500 mg IR [11], or NX-13 500 mg DR [11] and received at least one dose. There were no serious adverse events or deaths during the trial. One patient [500 mg DR, 1/11] withdrew due to worsening of UC and a second [500 mg IR, 1/11] on the last day of treatment after a panic attack associated with atrial fibrillation. In the efficacy population [36 patients], clinical improvement in rectal bleeding and stool frequency scores relative to placebo were seen as early as week 2 and endoscopic response was seen at week 4.

**Conclusions:**

NX-13 was generally safe and well tolerated with early signs of rapid symptom and endoscopic improvement. This novel mechanism of action warrants further investigation. ClinicalTrials.gov: NCT04862741.

## Graphical Abstract

**Graphical Abstract:**
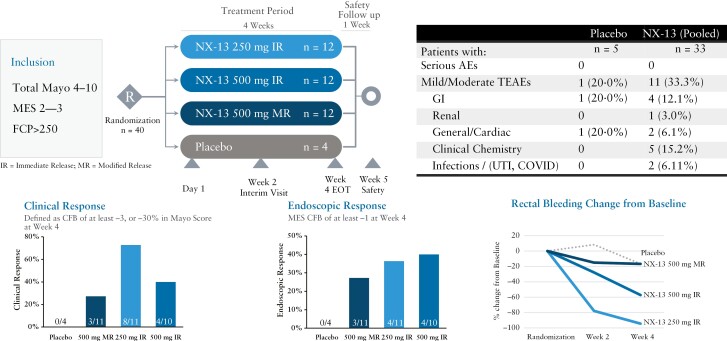

## 1. Introduction

Ulcerative Colitis [UC] is a chronic disabling inflammatory disease of the colon characterized by bloody diarrhoea and systemic symptoms. Despite progress in treatment over the last two decades with the approval of several monoclonal antibodies^1–5^ many patients do not respond to available agents or lose response over time. More recently, approval of oral small molecules such as Janus kinase [JAK] inhibitors^6,7^ and sphingosine-1-phospate receptor [S1P] modulators^8,9^ has expanded the available options. However, a therapeutic ceiling persists such that absolute rates of clinical remission are, at best, no more than 25% greater than with placebos.^10^ Safety concerns with existing mechanisms of action [MOAs] also underline the need to explore therapies with new treatment targets.^11,12^

NLRX1 (NOD [nucleotide oligomerization domain] like receptor X1) is a negative regulatory NOD-like receptor [NLR] which is present on the mitochondrial membranes of most cells including immune, epithelial, muscle and liver cells.^13^ The majority of NLRs, including *NOD2* which is genetically linked to Crohn’s disease [CD] and inflammasome-associated NLRs such as NLRP3, are pro-inflammatory.^14^ In contrast, NLRX1 is primarily immunoregulatory and inhibits, among others, nuclear factor kappa-light-chain-enhancer of activated B cells [NF-κB] and interferon-γ [IFNγ] inflammation.^13,15–17^ NLRX1 in immune and epithelial cells also modulates metabolic pathways and antioxidant enzyme expression to further enforce an anti-inflammatory phenotype.^18,19^ Activation of NLRX1 has the potential to be an effective treatment strategy to ameliorate the immune and metabolic dysfunction that characterizes active UC.^20–23^

NX-13 is a structurally designed triphenyl which is orally available with limited systemic absorption suggesting the potential to have a gut-selective distribution with low systemic exposure.^24,25^ Activation of the NLRX1 receptor in the bowel by NX-13 with associated improvement in chemical [DSS], cellular transfer [naïve CD4+ adoptive transfer] as well as genetic [*Mdr1a-/-*] models of inflammatory bowel disease [IBD] has been demonstrated.^26,27^ Mechanistically, NX-13 treatment induced NLRX1 expression, which upregulated oxidative phosphorylation gene expression while decreasing Th1/Th17 cell differentiation, neutrophil infiltration, and proinflammatory cytokines.^27^

In a previous phase 1 trial in healthy volunteers, NX-13 was given up to 4000 mg daily in single ascending dose [SAD] and multiple ascending dose [MAD] cohorts. It was well tolerated and safe, while displaying a gut-selective tissue distribution with most of the administered agent retrieved in the subject’s stool as compared to levels measured in the plasma.^24^ The clinical potential of locally acting mechanisms has been validated.^4^ We now report a phase 1b trial, which was designed with the primary aims of safety and confirmation of pharmacokinetics [PK] in patients with active UC.

## 2. Materials and Methods

### 2.1. Study design

**Figure 1.**
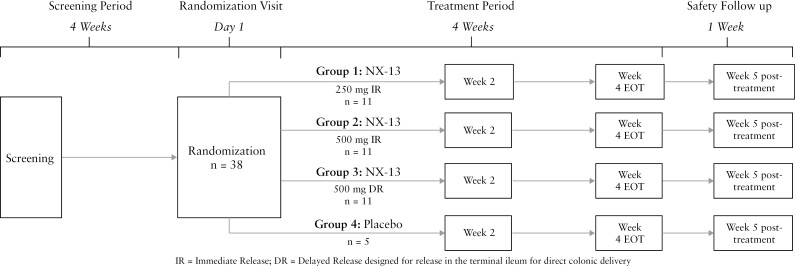
Design of the phase 1b trial: a randomized, double-blind study to evaluate the safety, tolerability, and pharmacokinetics of oral NX-13 in active ulcerative colitis. EOT: End of Treatment.

From April 2021 to June 2022, we conducted a multicentre, randomized, double-blind, placebo-controlled trial of NX-13 in patients with moderate active UC [ClinicalTrials.gov Identifier: NCT04862741]. A total of 24 community sites were recruited in the USA and Ukraine. The trial protocol was approved by a central Institutional Review Board, and all patients signed an informed consent form. The study consisted of a 4-week screening period [days –28 to 0], an eligibility review and randomization visit [day 1], followed by a 4-week treatment period [days 1–28] and a follow-up safety visit at week 5 [day 35] [Figure 1]. Data pertaining to exploratory endpoints were collected at screening, randomization, week 2, and week 4 visits to ascertain clinical efficacy, symptomatic relief, target engagement, and pharmacodynamic [PD] information [Supplementary Table 1]. Efficacy data were collected after 4 weeks of treatment for clinical endpoints including total Mayo Score, changes in faecal calprotectin [FCP], histology, and gene expression (mRNA and immunohistochemical [IHC] detection) while symptoms and FCP were collected at baseline, week 2, and week 4.

### 2.2. Participants

Key inclusion criteria included an endoscopically confirmed diagnosis of UC established for a minimum of 3 months before the screening visit. Eligible patients had active disease, defined as a total Mayo score (a four-component score comprising Rectal Bleeding Score [RBS, range 0–3], Stool Frequency Score [SFS, range 0–3], Mayo Endoscopic Score [MES, range 0–3], and Physician Global Assessment [PGA, range 0–3]) of 4–10, an MES of 2–3, and an FCP > 250 μg/g. The inclusion of patients with total Mayo scores in the range of 4–10 was chosen to support this phase 1b safety and PK study given that inclusion of patients in this range was not anticipated to impact the primary endpoints [safety, PK] of the trial.

Key exclusion criteria included diagnosis of CD or indeterminate disease, severe colitis [defined as per modified Truelove and Witts criteria], a history of non-response to two or more classes of biologics/advanced therapies, use of >20 mg prednisone [9 mg budesonide], and use of topical therapy with either 5-ASA or corticosteroids. The corticosteroid [≤20 mg/day prednisone, ≤9 mg/day budesonide, or equivalent] and 5-ASA [≤4.8 g/day] dose was required to be stable for 4 weeks prior to screening and throughout the trial.

Prior medications that required a washout period included intravenous or rectal corticosteroids [2 weeks], topical 5-ASAs [2 weeks], 6-mercaptopurine [25 days], azathioprine [25 days], cyclosporine [4 weeks], methotrexate [25 days], and biologics [8 weeks]. In addition, a 4-week washout was required for advanced small molecule therapy [i.e. tofacitinib, upadacitinib, ozanimod].

Endoscopies at screening and at week 4 were evaluated and scored by a central reader, who was blinded to the clinical disease activity scores, visit schedule, and the assigned treatment group of the patient.

### 2.3. Randomization

Patients were randomized using an electronic Interactive Web Response System [IWRS]. Eligible patients were randomly assigned in a centralized manner, in a 3:3:3:1 ratio, to receive either NX-13 250 mg IR, 500 mg, IR 500 mg DR, or placebo tablets to be taken one tablet orally once daily. Each of the NX-13 treatment groups comprised 11 patients and five patients were randomized to receive placebo.

### 2.4. Study procedures

Dosages of NX-13 for the phase 1b trial were selected based upon preclinical data. To assess the benefits of concentrated local drug exposure, a DR tablet was formulated to release NX-13 in the terminal ileum. All NX-13 and placebo tablets were supplied as identical-looking white, film-coated, capsule-shaped, biconvex tablets. NX-13 tablets used in this study were 250 and 500 mg IR and 500 mg DR all size- and weight-matched to placebo. The final label was coded for blinding as appropriate to the study design and in compliance with the protocol.

One tablet was taken orally once daily. Drug accountability, compliance checks, and diary data review were the responsibility of the principal investigator and clinical study coordinator.

### 2.5. Outcomes

The primary endpoint was safety and tolerability of the varied dosages of NX-13. This includes clinical adverse events [AEs], cardiac measures, vital signs, and laboratory parameters.

The secondary endpoints for PK analysis included NX-13 concentrations in colonic tissue, urine, plasma, and stool. PK and PD parameter estimates were derived using non-compartmental methods and calculated using a validated version of Phoenix WinNonlin [version 8.3].

Exploratory endpoints aimed to evaluate clinical efficacy, biomarkers, and PD effects of NX-13 [Supplementary Table 1]. These included mean change from baseline [CFB] in the Total [four-component] Mayo Score, and selected Mayo subscores [RBS, SFS, and MES] and histological remission [defined by Geboes score]. Colonic gene expression fold change was evaluated by quantitative real-time PCR [qRT-PCR]. Both endoscopy and histology were centrally read by a blinded reader. Based upon the results of the exploratory efficacy outcomes, post-hoc analyses were performed including evaluation of clinical response and remission, symptomatic remission, endoscopic response and remission, FCP CFB, and NLRX1 protein expression CFB. Analyses were performed on the Efficacy Analysis set, which included all patients in the Safety population [38] except two patients excluded for lack of diagnostic source documentation. The decision to exclude these patients from efficacy analyses was made before data unblinding due to observed irregularities in study operations at an individual site.

### 2.6. Biospecimen collection and processing

Plasma, stool, and urine were collected at randomization, week 2, and week 4 for PK analysis. Colonic biopsies were collected at baseline and week 4 from the most severely affected area of the colon.

### 2.7. Bioanalytical methods

NX-13 concentrations were measured using a liquid/liquid extraction, followed by high-performance liquid chromatography and eluate monitoring by an API 4000 MS/MS detector in positive multiple reaction monitoring mode. The methods were validated using an internal standard.

### 2.8. Histology and immunohistochemistry

Formalin-fixed colon biopsies were processed into paraffin blocks and used to assess cellular content by haematoxylin and eosin [H&E] staining and IHC for NLRX1 expression. A commercially available antibody for NLRX1 [Invitrogen MA5-27207; Clone: OTI4H8] was used. The H&E and IHC images were qualitatively scored by a blinded pathologist based on the Geboes score^28^ [H&E] and a scoring matrix considering both the NLRX1 intensity of staining as well as the proportion of cells which were positive for NLRX1 separately [NLRX1 IHC]. Lamina propria mononuclear cells [LPMCs] and intestinal epithelial cells [IECs] within each section were scored separately. A single composite mean NLRX1 score was then used due to the high correlation between these separate measures at baseline.

### 2.9. mRNA extraction and qRT-PCR method

Standard RNA extraction from RNAlater-preserved colon biopsies was performed by a contract laboratory. Standard Taqman qRT-PCR protocols were developed and validated, and final expression levels were calculated using a delta–delta Ct method to calculate fold-change in expression compared to baseline levels.

### 2.10. Statistical analysis

No formal sample size calculation was performed. A sample size of ~40 subjects, with 12 subjects randomized to each of the treatment groups and four subjects randomized to placebo, was considered sufficient to evaluate the safety endpoints and objectives of the study. A target recruitment of 38 patients was achieved.

Safety, PD parameters, and continuous clinical exploratory endpoints were summarized at each time point using the descriptive statistics of *N*, mean, standard deviation [SD], standard error [SE], median, and minimum and maximum values. Similar summaries were presented for CFB values. Mean and individual plasma concentration–time curves of NX-13 were tabulated for each dose cohort. In addition, geometric means were calculated for the area under the curve and maximum concentration [Cmax]. Categorical safety and clinical endpoints were summarized as counts and proportions.

### 2.11. Role of the funding source

This study was funded by Landos Biopharma, Inc., Blacksburg, VA, USA. The sponsor offered input towards the study design; collection, analysis, and interpretation of the data; and preparation, review, and approval of the manuscript. The authors had full access to the data and control of the final approval and decision to submit the manuscript.

## 3. Results

### 3.1. Patients

**Figure 2.**
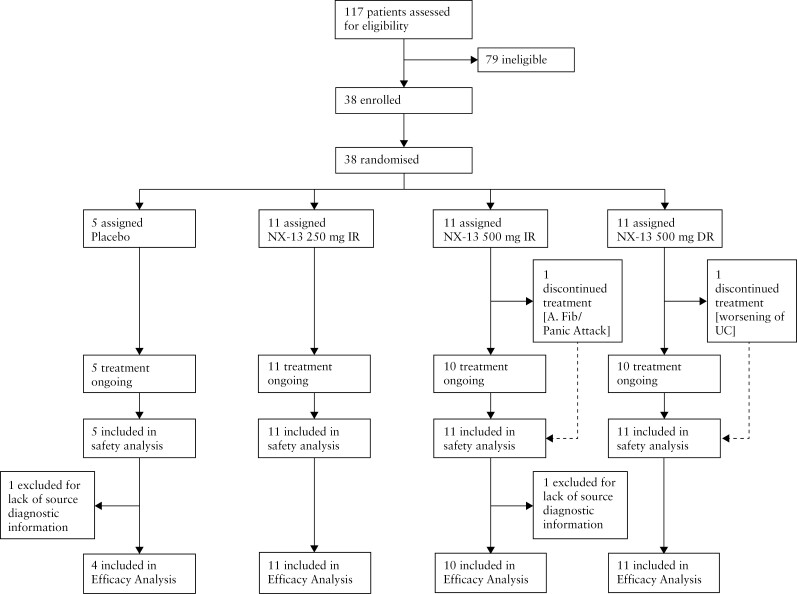
Profile of the phase 1b trial accounting for all enrolled and evaluated patients.

Thirty-eight patients [11 females] with active UC were recruited and randomized to placebo [*n* = 5], NX-13 250 mg IR [*n* = 11], NX-13 500 mg IR [*n* = 11], or NX-13 500 mg DR [*n* = 11] from April 26, 2021, to May 16, 2022 [Figure 2]. The baseline characteristics of the 36 patients in the efficacy analysis are shown in Table 1. Mean total Mayo scores were similar across groups with a slight elevation in the 500 mg DR group [Table 1]. Endoscopic scores were also similar. Most patients were receiving aminosalicylates at entry, with a lower percentage of patients receiving stable corticosteroids. Four [of 36] patients had previous biologic exposure [one patient in each dose group and placebo].

**Table 1.** NX-13 phase 1b trial demographics and baseline characteristics for the efficacy analysis set [*n* = 36].

	Placebo [n = 4]	NX-13 250 mg IR [n = 11]	NX-13 500 mg IR [n = 10]	NX-13 500 mg DR [n = 11]	All patients [n = 36]
Sex					
Male	1 [25%]	11 [100%]	5 [50%]	10 [90.9%]	27 [76%]
Female	3 [75%]	0 [0%]	5 [50%]	1 [9.1%]	9 [24%]
Geography					
USA	2 [50%]	5 [45%]	5 [50%]	6 [55%]	18 [50%]
Ukraine	2 [50%]	6 [55%]	5 [50%]	5 [45%]	18 [50%]
Age [years]	49.2 [18.0]	50.5 [14.4]	54.9 [15.2]	42.6 [15.5]	49.3 [15.5]
Total Mayo	7.3 [1.5]	7.0 [1.7]	7.3 [1.9]	8.6 [1.8]	7.6 [1.8]
RBS	1.75 [0.96]	1.18 [0.60]	1.30 [0.67]	1.45 [0.52]	1.36 [0.64]
SFS	2.00 [0.82]	1.45 [1.29]	1.60 [1.07]	2.36 [0.81]	1.83 [1.08]
MES	2.50 [0.58]	2.45 [0.52]	2.30 [0.82]	2.55 [0.52]	2.44 [0.61]
FCP [µg/g]	1608.1 [1339.21]	1563.2 [2178.46]	1603.0 [1327.33]	1216.5 [1952.55]	1480.6 [1748.89]
Geboes score	7.7 [6.66]	11.3 [6.50]	13.1 [6.94]	9.3 [6.02]	10.9 [6.45]
Baseline 5-ASA use	2 [50%]	8 [73%]	7 [70%]	7 [64%]	24 [67%]
Baseline steroid use	1 [25%]	2 [18%]	0 [0%]	3 [27%]	6 [17%]
Previous biologic use	1 [25%]	1 [9·1%]	1 [10%]	1 [9·1%]	4 [11%]

Data are *n* [%], mean [SD]. RBS, Rectal Bleeding Score; SFS, Stool Frequency Score; MES, Mayo Endoscopic Score. Geboes score represents the continuous Geboes calculation. IR, immediate release; DR; delayed release; FCP, faecal calprotectin.

#### 3.1.1. Safety

NX-13 was generally safe and well tolerated in all patients studied [Table 2]. No deaths nor serious adverse events [SAEs] were reported. All the AEs were mild to moderate. There were two cases of infections that did not require drug withdrawal: a urinary tract infection [250 mg IR] and a COVID-19 infection [500 mg DR]. One patient who received 500 mg DR was withdrawn for worsening disease and one patient assigned to placebo experienced worsening of UC, dizziness, and weakness. In two patients [500 mg DR] with pre-existing hyperlipidaemia at screening, an AE of hyperlipidaemia was reported. One patient assigned to 500 mg DR reported mild abdominal pain on day 1 which resolved the next day. In the 500 mg IR group one patient experienced a panic attack on the last study day pre-dosing. Thirty minutes later a transient episode of atrial fibrillation was reported and was deemed related to the panic attack. Sinus rhythm converted back to normal in a few hours spontaneously. A cardiology assessment occurred the next day and further follow-up was not recommended. One patient assigned to 500 mg DR completed 4 weeks of treatment without any AEs but due to the war in Ukraine did not return to the 1-week follow-up visit. However, he returned to the clinic 3 months after the last dose with elevated creatinine levels and suspected pancreatitis [no confirmatory test results provided]. The patient was treated for pancreatitis and sent home.

**Table 2.** NX-13 phase 1b adverse events in the safety analysis set [*n* = 38] by system organ class.

Patients with:	Placebo [n = 5]	NX-13 250 mg IR [n = 11]	NX-13 500 mg IR [n = 11]	NX-13 500 mg DR [n = 11]
Serious AEs	0	0	0	0
Mild/moderate TEAEs	1 [20.0%]	2 [18.2%]	3 [27.3%]	6 [54.5%]
GI [abdominal pain, UC worsening, pancreatitis, constipation]	1 [20.0%]	0	1 [9.1%]	3 [27.3%]
Renal [congenital PCKD, increased creatinine, kidney stones]	0	0	0	1 [9.1%]
General/cardiac [A. fib, anaemia, weakness, dizziness]	1 [20.0%]	0	2 [18.2%]	0
Clinical chemistry [GGT increase, HLD, hypocalcaemia, hypophosphataemia]	0	1 [9.1%]	2 [18.2%]	2 [18.2%]
Infections [UTI, COVID]	0	1 [9.1%]	0	1 [9.1%]

Data are *n* [%]. AEs, adverse events; TEAEs, treatment-emergent AEs; GI, gastrointestinal; PCKD, polycystic kidney disease; A. fib, atrial fibrillation; GGT, gamma glutamyl transferase; HLD, hyperlipidaemia; UTI, urinary tract infection; COVID, coronavirus disease 2019.

#### 3.1.2. Pharmacokinetics

**Figure 3.**
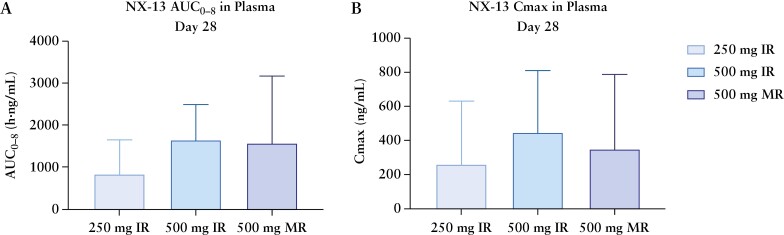
Pharmacokinetic measures of NX-13. Area under the curve [A] and Cmax [B] after 4 weeks of treatment.

The IR dose exhibited low plasma PK levels which peaked at 1 h, while the DR dose was absorbed later and was maintained a low exposure. After 28 days, exposure was approximately dose proportional between the IR dose groups, with Cmax of the 500 mg DR group intermediate between that of the 250 and 500 mg IR doses [Figure 3]. All NX-13 patients experienced an NX-13 concentration decline in a multi-exponential fashion. There was no evidence of accumulation of NX-13 during the study. As expected, NX-13 500 mg DR resulted in higher concentrations in stool compared to the IR doses. NX-13 was not found in the urine. Although patients in the higher dose groups had measurable tissue levels, overall trends in NX-13 tissue exposure were not evaluable, and a more sensitive detection assay will be developed.

#### 3.1.3. Exploratory clinical endpoints

**Figure 4.**
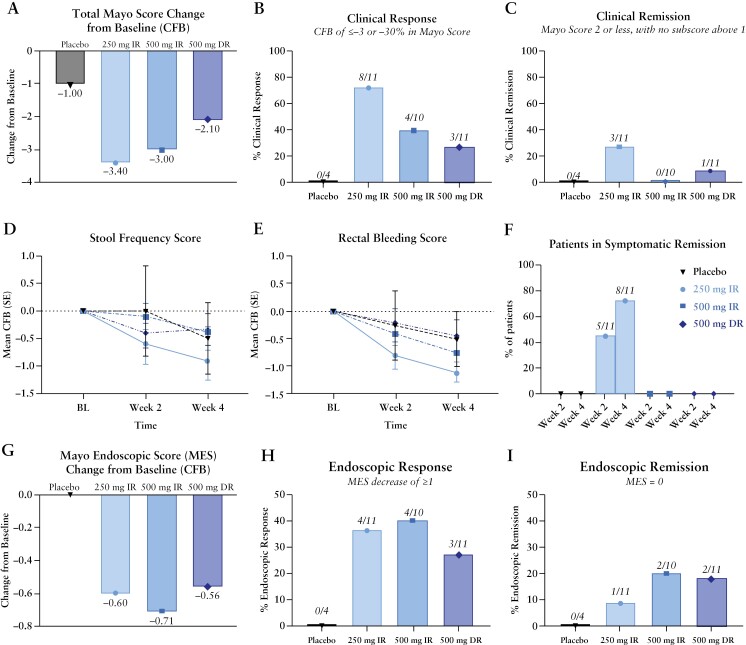
Clinical outcomes of patients treated with NX-13 or placebo. NX-13-treated patients experienced reductions in total Mayo score [A] and higher rates of clinical response [B] and clinical remission [C] after 4 weeks. Patient-reported outcomes showed NX-13-treated patients experienced reductions in SFS [D], RBS [E], and symptomatic remission, defined as RBS = 0, SFS = 0 [F] after 2 and 4 weeks. Endoscopic outcomes showed NX-13-treated patients experienced reductions in MES [G], and higher rates of endoscopic response [H] and endoscopic remission [I] after 4 weeks. SFS, Stool Frequency Score; RBS, Rectal Bleeding Score; MES, Mayo Endoscopic Score.

The trial was not powered for the exploratory clinical endpoints; therefore, the clinical results are descriptive. Clinical improvement was observed for the majority of patients treated with NX-13 for 4 weeks. Patients who were treated with the 250 mg IR dose had the greatest average reduction in total Mayo score [Figure 4A], with a mean decrease of 3.4 points [range −7.0 to 0] which represented a 49% reduction after 4 weeks. Patients treated with 500 mg IR achieved a mean reduction of 3.0 [range −6.0 to +1.0], and those treated with 500 mg DR had a mean reduction of 2.1 [range –8.0 to +4.0]. In contrast, the placebo total Mayo score improvement at 4 weeks was 1.0 point [−2 to 0]. Clinical response at 4 weeks was achieved in 8/11 patients treated with 250 mg IR, 4/10 of patients treated with 500 mg IR, and 3/11 treated with 500 mg DR [Figure 4B] compared to none [0/4] of the patients on placebo. At 4 weeks, clinical remission was achieved in 3/11 of the patients on 250 mg and a single patient treated with 500 mg DR [Figure 4C].

Improvement in symptoms [decrease in RBS or SFS] was achieved by the majority of patients across all NX-13 dose groups [Figure 4D-F]. The low-dose group responded quickly with 6/11 having an SFS of 0 already at week 2, and 8/11 reported an SFS of 0 at 4 weeks. This equated to an average decrease in SFS of 58% at week 2 and 72% at week 4 in the 250 mg IR group [Figure 4D]. The 500 mg IR group’s average SFS improvement at 4 weeks was similar to those in the 500 mg DR and placebo groups [Figure 4D]. None of the placebo or 500 mg DR patients achieved an SFS of 0 during the trial. RBS improvement was also marked and rapid in the NX-13-treated groups compared to placebo [Figure 4E]. Again, the 250 mg IR group saw the greatest improvement with 78% and 94% decreases in RBS at 2 and 4 weeks, respectively [Figure 4E]. This was reflected in the majority of 250 mg IR patients who had no blood in their stool: 6/11 at 2 weeks and 9/11 at 4 weeks. While less pronounced, the 500 mg IR dose group also showed improvement as 3/10 and 4/10 of patients in the 500 mg IR group achieved an RBS of 0 at 2 and 4 weeks, respectively. Improvement in the 500 mg DR group’s average RBS was similar to placebo at weeks 2 and 4 of treatment. None of the placebo patients achieved an RBS of 0 during the trial. Complete resolution of both diarrhoea and rectal bleeding was achieved at 2 weeks in 5/11 of patients treated with 250 mg IR which increased to 8/11 at week 4 [Figure 4F].

Patients treated with NX-13 also experienced endoscopic improvement during the 4-week treatment period. All NX-13 doses showed CFB in MES at 4 weeks [Figure 4G]. Endoscopic response [Figure 4H] was achieved in 4/11 of patients treated with 250 mg IR, 4/10 of patients treated with 500 mg IR, and 3/11 treated with 500 mg DR. None of the four patients randomized to placebo achieved endoscopic response. Endoscopic remission was achieved in 5/32 patients treated with active agent after 4 weeks of treatment [Figure 4I]. There was a close correlation between endoscopic improvement and patient-reported outcomes [PROs], as nearly all the endoscopic responders [10/11] showed a rapid onset of symptomatic improvement in either RBS or SFS already at week 2. Further, all five of the endoscopic remitters had symptomatic improvement at week 2.

Histological remission was also seen in patients across dose groups in similar ratios to endoscopic response [Supplementary Figure 1], although the one placebo patient who reached histological remission was not histologically active at baseline so did not demonstrate improvement but maintenance of remission. Histological improvement was also supported by CFB in continuous Geboes calculations [data not shown]. Consistent with clinical improvement there was a mean decrease in FCP concentration [Supplementary Figure 2] in both the 250 and 500 mg IR groups which was not observed in the 500 mg DR or placebo-treated groups. FCP improvement also correlated with clinical response status [Supplementary Figure 2B], with numerically greater reductions in FCP in those IR patients who responded than in those who did not respond.

#### 3.1.4. Exploratory pharmacodynamic endpoints

**Figure 5.**
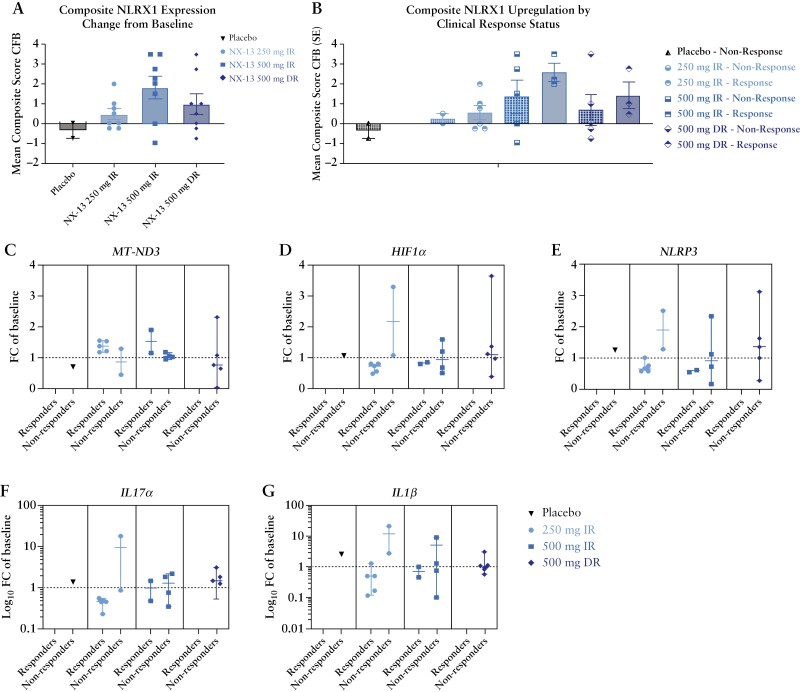
Pharmacodynamic observations in patients treated with NX-13 or placebo. Qualitative NLRX1 expression in colon tissue of patients was increased in patients treated with NX-13 [A] and correlated with patients’ clinical response [B]. NX-13 induced changes in expression of metabolic and immune signalling genes *MT-ND3* [C], *HIF1A* [D], *NLRP3* [E], *IL17A* [F], and *IL1b* [G] expression in colon tissue which correlated with clinical response after 4 weeks of treatment.

NX-13-treated patients demonstrated approximately exposure-proportional increases in NLRX1 qualitative IHC scores in colonic tissue biopsies collected at week 4 compared to baseline (Figure 5A). Samples from placebo-treated patients did not show increases in NLRX1 expression. Interestingly, within each dose group, patients in clinical response tended to show visually greater upregulation of NLRX1 levels, although the number of patients does not permit statistical analysis [Figure 5B]. Increases in NLRX1 IHC staining were observed consistently in both IECs and LPMCs.

We next explored the gene expression profiles in colonic biopsy samples after 4 weeks of NX-13 or placebo treatment and compared these to baseline expression. *MT-ND3* upregulation was most pronounced in clinical responders in the IR-treated groups [Figure 5C]. NLRX1 has been shown to affect intracellular signalling cascades including NFκB and HIF1α.^27^*HIF1α* downregulation was more common in responders in the IR groups, suggesting a role in response [Figure 5D]. NLRP3 is a pro-inflammatory member of the inflammasome complex responsible for caspase-1 cleavage and downstream activation of pro-IL-1β.^29^*NLRP3* was downregulated in the IR dose groups with a similar visual correlation of downregulation in patients in clinical response [Figure 5E]. Both *IL-17α* and *IL-1*β were downregulated about 50% in the 250 mg IR group, with visual trends observed between expression and clinical response [Figure 5F–G].

## 4. Discussion

This phase 1b trial of NX-13, which represents a new class of immunometabolic modulators, shows that the compound was safe and well tolerated. No SAEs were reported. Moderate AEs of worsening of UC and atrial fibrillation led to withdrawal of two patients from the trial. Although AEs were seen more frequently with the higher DR dose of the drug, those patients also had higher disease activity at baseline. The short duration of the trial precluded evaluation of long-term safety which will be further evaluated in phase 2 studies.

NX-13 has potential to be gut-selective with low systemic exposure as compared to the gut-restricted exposure seen preclinically. In preclinical pig studies, plasma concentrations were <0.5% compared to those in the stool, and only one-tenth of tissue concentrations which suggested limited absorption, supporting the characterization as a gut-restricted molecule in preclinical species [Landos Biopharma, unpublished data]. However, the results of the human clinical trials and the superior clinical performance of the IR doses support the potential relevance of the low systemic exposure. Importantly, the level of systemic exposure observed does not appear to compromise the safety of NX-13, as the IR doses were not associated with an increased risk of AEs. It is noteworthy that the minimal systemic exposure observed may be desirable for the use of NX-13 in transmural intestinal disorders such as CD.

Although the study was not powered for clinical endpoints, total Mayo scores showed consistent, rapid, and clinically meaningful improvement from baseline to week 4 in all three treatment groups. These changes were not observed in the placebo group [Figure 4]. The 250 mg IR group displayed the greatest improvement. There was substantial improvement in PROs: rectal bleeding and diarrhoea were both reduced in NX-13 IR patients to a greater degree than placebo [Figure 4]. Indicative of rapid healing was the fact that in the 250 mg IR group, 5/11 at week 2 and 8/11 at week 4 reported no rectal bleeding or diarrhoea, although it should be mentioned that the average baseline RBS and SFS were lowest in the 250 mg IR group compared to the other groups. The objective measure of MES also improved in all NX-13-treated groups [Figure 4], and there was a concurrence of endoscopic response and PRO improvement. Finally, further consistency of response was seen in the histological remission rates and reduction in FCP levels. Given the 4-week duration of the trial we theorize that further clinical and endoscopic improvement would continue with prolonged drug exposure. This concept will be evaluated in future trials.

Signals of target engagement as measured by the increased NLRX1 expression in the colon in both IECs and LPMCs showed that local colonic concentrations were sufficient to induce NLRX1 and downstream immune and metabolic signals consistent with those observed in preclinical studies.^27^ NLRX1 mRNA expression levels displayed a similar increase in NX-13-treated patients as protein expression [Supplementary Figure 3] although to a lesser extent. However, it is not known if NLRX1 upregulation by NX-13 is primarily a gene expression-, mRNA stability-, or protein-based [stability or degradation] event.^27^

Interestingly, the 250 mg dose group had the best clinical response in this trial, although it did not produce the greatest increase in NLRX1 levels. Future studies will be designed to further define the dose–response relationship and define PK/PD relationships. Additionally, we plan to improve the sensitivity of our drug assay to facilitate these assessments. Nonetheless, such strong signals of efficacy in the 250 mg IR dose warrant its inclusion in later trials.

The potential to treat UC immunometabolically to affect not just the overt immune response, but also the underlying metabolic homeostasis of the immune and epithelial cells shows promise. In both immune and epithelial cells from IBD patients, mitochondrial metabolism genes account for the majority of downregulated genes relative to healthy controls, and various studies have validated the importance of epithelial cell and immune cell mitochondrial function in animal models of IBD.^20,22,23^ Even so, the metabolic and mitochondrial deficiencies in UC, and other gastrointestinal inflammatory diseases, remain largely unaddressed by current treatment paradigms. Understanding the relationship between baseline metabolic characteristics, successful immunometabolic modulation, and the rapidity of clinical response will be a focus of future clinical and translational work.

This was a phase 1b trial, which led to some limitations. The clinical endpoints were exploratory or post-hoc analyses and are hypothesis-generating only, although the concordance with preclinical outcomes is encouraging. There were 35 patients who concluded the trial, with 4/35 in the placebo subgroup. Although we anticipated 40% of patients studied to have been biologic-experienced, only 11% had previously failed a biologic which limits our ability to claim any efficacy in this subpopulation. The predominance of bionaive patients recruited could be due to the majority of sites being community centres as well as 50% of the trial participants recruited from eastern Europe where access to advanced therapies can be limited. Most patients were 5-ASA failures. However, inclusion was restricted to patients with an MES of 2–3, ensuring that significant disease was present at baseline. Safety data were collected for 5 weeks. PK analysis did not reveal consistent NX-13 tissue concentration levels and a more sensitive detection assay will be developed to clarify the site and mechanism of action of NX-13. Similarly, regarding the PD results, we reiterate that while they suggest positive target engagement, interpretation is limited by the small trial size and the number of patients whose samples were available for analysis.

In this phase 1b trial, NX-13 was safe and well tolerated. Dosing with NX-13 resulted in rapid and robust clinical, endoscopic, histological improvement by week 4 with evidence of early symptom and biomarker improvement as early as 2 weeks. Encouraged by the observations in this phase 1b study, NX-13 is currently under investigation in a randomized, placebo-controlled phase 2 proof-of-concept study [NCT05785715].

## Supplementary Material

jjad192_suppl_Supplementary_Materials

## Data Sharing

These studies were preregistered with an analysis plan at ClinicalTrials.gov [NCT04862741]. Upon request, and subject to review, Landos will provide the data that support the findings of this study.
