# Supplementary material for: The Safety, Tolerability, Pharmacokinetics, and Clinical Efficacy of the NLRX1 agonist NX-13 in Active Ulcerative Colitis: Results of a Phase 1b Study
Source: J Crohns Colitis. 2023 Nov 11;18(5):762–72. doi: 10.1093/ecco-jcc/jjad192 (PMC11140628; doi:10.1093/ecco-jcc/jjad192)
Supplement: jjad192_suppl_Supplementary_Materials [file jjad192_suppl_supplementary_materials.pdf]

TITLE: The Safety, Tolerability, Pharmacokinetics and Clinical Efficacy of the NLRX1 agonist NX-13 in Active Ulcerative Colitis: Results of a Phase 1b Study

Authors: Bram Verstockt, Severine Vermeire, Laurent Peyrin-Biroulet, et al.

Supplementary Tables and Figures

Endpoint class	Outcome	Definition
Exploratory	Change in Total Mayo	Mean change in Mayo Score from baseline to Week 4
Exploratory	Change in Rectal Bleeding Score (RBS)	Mean change in the Mayo subscore of rectal bleeding from baseline to Weeks 2 and 4
Exploratory	Change in Stool Frequency Score (SFS)	Mean change in the Mayo subscore of stool frequency from baseline to Weeks 2 and 4
Exploratory	Change in Mayo Endoscopic Score (MES)	Mean change in MES from baseline to Week 4
Exploratory	Histologic Remission	Percentage of patients with histological remission, defined as Geboes score < 3.1 with absence of increased neutrophils in the lamina propria, at Week 4
Exploratory	Colonic Tissue Gene Expression	Fold Change over baseline value of NLRX1, MT-ND3, HIF1α, NLRP3, IL-17α, IL-1β in colon tissue biopsies
Post-Hoc	Clinical Remission	Total Mayo Score of <2 with no subscore >1
Post-Hoc	Clinical Response	Decrease in Total Mayo Score of ≥3 or ≥30%
Post-Hoc	Symptomatic Remission	Scores of 0 for BOTH RBS and SFS
Post-Hoc	Endoscopic Remission	Mayo Endoscopic Score (MES)=0
Post-Hoc	Endoscopic Response	Decrease in MES of ≥1
Post-Hoc	NLRX1 Expression Levels	Change from baseline in Qualitative expression score measured by centrally read IHC in colonic tissue biopsies
Post-Hoc	Change in Fecal Calprotectin	Mean change in fecal calprotectin from baseline (randomization) to Weeks 2 and 4

Supplemental Table 1: Clinical endpoints and definitions used in exploratory and post-hoc analyses.

Gene Symbol	Gene
ACTB	Beta-actin
GPX1	Glutathione peroxidase
HIF1a	Hypoxia-inducible factor 1-alpha
IL-10	Interleukin-10
IL-17a	Interleukin-17 alpha
IL-1b	Interleukin-1 beta
IL-23a	Interleukin-23 alpha
ITGAE	Integrin-alpha-E
ITGB7	Integrin-beta-7
MT-ND3	Gene of the mitochondria genome coding for the NADH dehydrogenase
NLRP3	NLR family pyrin domain containing protein 3
NLRX1	NOD [Nucleotide Oligomerization Domain] Like Receptor X1
ODGH	Oxoglutarate dehydrogenase
RPS18	Ribosomal protein S18
SIRT1	NAD-dependent deacetylase sirtuin-1
TJP1	Tight junction protein ZO-1
TNF	Tumor necrosis factor alpha
TXNRD1	Thioredoxin reductase-1

Supplemental Table 2: Complete panel of genes screened for changes in gene expression in colonic biopsies.

Supplemental Figure 1: NX-13 induces or maintains Histological Remission after 4 weeks as measured by the Geboes Score.

Supplemental Figure 2: Fecal Calprotectin levels measured after 4 weeks of treatment with NX-13 show a negative mean change from baseline (A) and a greater reduction in responders compared to non-responders (B).

Supplemental Figure 3: NX-13 induces modest upregulation of the NLRX1 transcript in colonic tissue.

Supplemental Figure 1

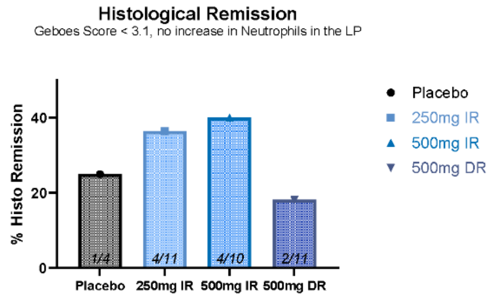

Supplemental Figure 1: NX-13 induces or maintains Histological Remission after 4 weeks as measured by the Geboes Score

Supplemental Figure 2

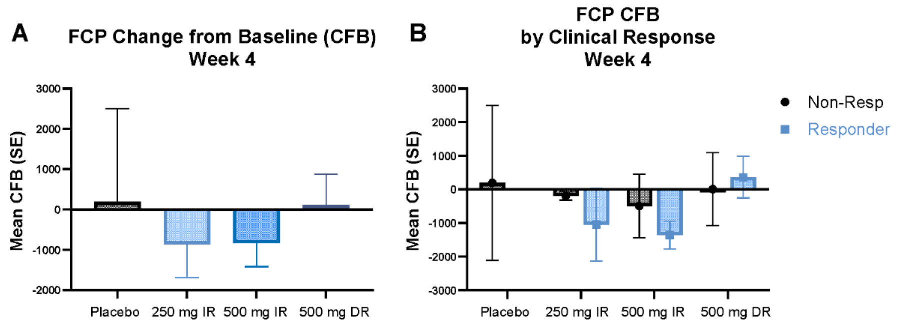

Supplemental Figure 2: Fecal Calprotectin levels measured after 4 weeks of treatment with NX-13 show a negative mean change from baseline (A) and a greater reduction in responders compared to non-responders (B).

Supplemental Figure 3

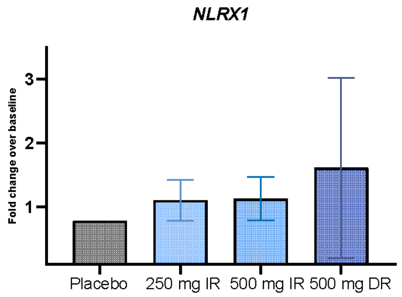

Supplemental Figure 3: NX-13 induces modest upregulation of the NLRX1 transcript in colonic tissue.
